# Survival of low birthweight neonates in Uganda: analysis of progress between 1995 and 2011

**DOI:** 10.1186/s12884-018-1831-0

**Published:** 2018-05-30

**Authors:** Malachi Ochieng Arunda, Anette Agardh, Benedict Oppong Asamoah

**Affiliations:** Social Medicine and Global Health, Department of Clinical Sciences, Lund University, Jan Waldenströms gata 35, 205 02 Malmö, Sweden

**Keywords:** Low birthweight, Attributable neonatal mortality, Logistic regression, Kaplan-Meier survival analysis, Cross-sectional

## Abstract

**Background:**

Although low birthweight (LBW) babies represent only 15.5% of global births, it is the leading underlying cause of deaths among newborns in countries where neonatal mortality rates are high. In Uganda, like many other sub-Saharan African countries, the progress of reducing neonatal mortality has been slow and the contribution of low birthweight to neonatal deaths over time is unclear. The aim of this study is to investigate the association between low birthweight and neonatal mortality and to determine the trends of neonatal deaths attributable to low birthweight in Uganda between 1995 and 2011.

**Methods:**

Cross-sectional survey datasets from Uganda Demographic and Health Surveys between 1995 and 2011 were analyzed using binary logistic regression with 95% confidence interval (CI) and Kaplan-Meier survival analysis to examine associations and trends of neonatal mortalities with respect to LBW. A total of 5973 singleton last-born live births with measured birthweights were included in the study.

**Results:**

The odds of mortality among low birthweight neonates relative to normal birthweight babies were; in 1995, 6.2 (95% CI 2.3 −17.0), in 2000–2001, 5.3 (95% CI 1.7 −16.1), in 2006, 4.3 (95% CI 1.3 − 14.2) and in 2011, 3.8 (95% CI 1.3 − 11.2). The proportion of neonatal deaths attributable to LBW in the entire population declined by more than half, from 33.6% in 1995 to 15.3% in 2011. Neonatal mortality among LBW newborns also declined from 83.8% to 73.7% during the same period.

**Conclusion:**

Low birthweight contributes to a substantial proportion of neonatal deaths in Uganda. Although significant progress has been made to reduce newborn deaths, about three-quarters of all LBW neonates died in the neonatal period by 2011. This implies that the health system has been inadequate in its efforts to save LBW babies. A holistic strategy of community level interventions such as improved nutrition for pregnant mothers, prevention of teenage pregnancies, use of mosquito nets during pregnancy, antenatal care for all, adequate skilled care during birth to prevent birth asphyxia among LBW babies, and enhanced quality of postnatal care among others could effectively reduce the mortality numbers.

## Background

About 20 million low birthweight (LBW) babies are born every year, representing 15.5% of all births globally [1]. Over 95% of all LBW cases occur in low-income countries [1]. Of recent, Lawn et al. and the World Health Organization (WHO) estimated that LBW contributes to 60–80% of all neonatal deaths (death within 28 days after birth) worldwide [2, 3]. However, wider disparities in estimates exist between countries. India, a low-to-middle income country, contributes about 40% of global burden of LBW babies [4], and in 2013, 48% of all neonatal deaths in India were attributed to LBW and preterm birth [5]. In comparison to Sweden, a high-income country where neonatal mortality is very low (1.5 per 1000 live births in 2014) [6], LBW babies constituted only 3.2% of national live birth in 2014, and barely 4.3% of all neonatal deaths in 2014 were LBW cases [6]. WHO defines LBW as birthweight of less than 2500 g [1]. LBW is mainly a result of preterm births and restricted fetal growth (resulting in small for gestational age (SGA) babies) or both [1]. The main risk factors leading to LBW include young mothers/short stature of the mother [7], multiple births [8], poor nutrition before conception and during pregnancy (poverty) [9], smoking [10], maternal HIV positivity, and malaria during pregnancy [11, 12].

In sub-Saharan Africa (SSA), the general rate of decline in neonatal mortality (NM) has been slow compared to infant or under-five mortality [13] and more than half of all births do not take place in health facilities [14]. An individual participant level meta-analysis study in four district projects within East Africa (EA) in 2012 estimated that 52% of all neonatal deaths in Kenya, Uganda, and Tanzania were attributable to preterm birth or small for gestational age, of which 99% were LBW babies [15]. Several neonatal and infant mortality studies in SSA fall short of determining the contribution of LBW to neonatal deaths. Whereas LBW is the underlying cause of majority of neonatal deaths, most studies have focused on other leading direct causes of neonatal deaths such as birth asphyxia, infections, and preterm birth [16–18]. Another 5-year health facility-based study in Ghana estimated that LBW was a sole contributor of 50% of neonatal deaths in the facility between 2008 and 2012 [19]. While LBW can be a result of preterm birth, it is also a notable fetal risk factor for birth asphyxia and infections such as sepsis [17, 18].

In Uganda, like in many SSA settings, apart from health system limitations such as inadequate resources, paucity of data in hospital registries makes it difficult to determine the prevalence of LBW and associated mortality trends [20, 21]. The 2008 situation analysis report indicated that neonatal deaths were not registered in Uganda; no countrywide perinatal audit exists [20]. The 2006 retrospective demographic survey in Uganda estimated that 60% of newborn deaths occurred at home [22]. The Uganda roadmap for reducing neonatal mortality 2007–2015 fell short of incorporating LBW among the causes of neonatal deaths [21], possibly due to challenges in determining LBW-attributable deaths. No studies that determined the national trends of LBW-attributable neonatal mortality in Uganda were identified by our literature search, despite being a key indicator of population and reproductive health in a country [2]. However, in order for Uganda to achieve the global Sustainable Development Goal (SDG) target 3.2 that aims to drastically reduce neonatal mortality by 2030 [23], the contribution of LBW towards neonatal mortality can no longer remain unclear. Although LBW is estimated to contribute about 80% of neonatal deaths in SSA [3], efforts to reduce neonatal mortality from the inception of the Millennium Development Goals (MDGs) in 1990 to its end in 2015 in Uganda have never been evaluated in terms of reduction of LBW-attributable deaths. Further, there are no national representative studies that have examined the contribution of LBW toward the overall neonatal mortality in Uganda. This present study thus aims to determine both the association between LBW and neonatal mortality in Uganda and to estimate the national trends of LBW-attributable neonatal mortality between 1995 and 2011. This period covered the entire MDG period except for the last 4 years to 2015.

## Methods

### Study setting and maternal health situation

With an annual population growth rate of about 3.2 and an overall fertility rate of 5.6, Uganda’s population rose from about 17 million in the 1990s to about 34 million in 2011 [24]. The sex ratio is 1:1 and the adolescence fertility rate was about 131 per 1000 births in 2010 [25]. Over 77% of the population live in rural areas. The national poverty levels notably reduced from 38.8% in 2002–2003 to about 20% in 2012–2013 [26]. However, poverty levels differ significantly by region and sub-regions. For instance, while incidence of poverty in the northern region in 2013 was 44%, it was only 5.1% in the central region [26]. In March 2001, Uganda abolished user fees in first level government health facilities and this included maternal health services [27]. The proportion of four or more antenatal care visits was still less than 50% by 2011 [28]. Incrementally, by 2011 about 57% of total births took place in health facilities and the proportion of births that received post-natal care increased from less than 10% in 1995 to 26% in 2006 and to 32% in 2011 [28, 29].

### Study design and data source

We obtained secondary data from repeated cross-sectional surveys by the Demographic and Health Survey (DHS) program. The datasets are independent and nationally representative. We used four datasets from the Uganda DHS birth recodes for the years 1995, 2000–2001, 2006, and 2011. A total of 5973 singleton last-born live births with birthweight measures were included in the study. This consisted of 1160 children in 1995 representing 25% of all the last-born live births in the data sample for that year and 1100 children for the year 2000–2001 representing 30% of all the last-born live births in the sample for that year. Similarly, 1514 (35%) children were included for the year 2006 and 2199 (50%) for the year 2011. We targeted and utilized the birth recode information for the last-born live births born within the 5-year period prior to each of the surveys. The Demographic and Health Survey (DHS) program employs standardized questionnaires and protocols that ensure that the participants remain anonymous [30, 31]. The DHS data collection procedure involves stratified two-stage cluster sampling and collection of data countrywide using updated lists of enumeration areas for each of the surveys to avoid overlap and improve national representativeness of the data [32]. Further information on data sampling and collection criteria are detailed in the DHS field manuals and methodology toolkits [30–32].

### Variables

#### Outcome variable

##### Neonatal mortality

This referred to death of newborn within 28 days after birth. It was dichotomized into yes (died) or no (alive).

### Predictor variable

#### Low birthweight

The variable low birthweight (LBW) was the predictor variable. Birthweight records were obtained from the child’s health card or from the mother’s verbal report of measured weight at birth. Birthweight was dichotomized into LBW (< 2500 g) or normal birthweight (NBW) ≥ 2500 g. Macrosomia (> 4000 g) [33] was eliminated in the univariate and logistic regression analyses involving birthweights. The higher neonatal mortality risks of macrosomia relative to NBW [34] would reduce the accuracy of our findings if they are included among NBW numbers. At the hospital, newborns are weighed and their birthweights recorded on the child’s health card and is communicated. In contrast, for births outside the health facility such as home births, birthweight is likely to be estimated by observing the birth size of body parts, the accuracy of which is questionable. To improve the accuracy of reported birthweight, whether recall or from the health card, only hospital births were included in the study for the years 2000−2001, 2006, and 2011. For the 1995 dataset, however, we also included the very few home birth cases in our sample in order to improve the statistical power of our analyses. Records of the size of the babies registered as small or average among others were excluded from the study to minimize errors of misclassification due to the unreliable subjective nature of the categorization criteria [35]. From the study’s selected samples, 72% of the 1160 selected sample in 1995 had birthweight from mothers’ recall and the rest were from health cards. Similarly, in 2000, 79% of the 1100 selected sample were from recall. In 2006, 73.5% of the total 1514 were recall birthweights and in 2011, 67% of the total 2199 were recall birthweights.

Preterm birth, LBW and birth asphyxia are highly correlated and it is difficult to determine their independent contributions towards neonatal deaths. These three, together with infections, contribute to 80% of neonatal mortality as the highest cause of neonatal mortality, with LBW being the underlying factor [36].

### Maternal and socio-demographic variables

**Fig. 1:**
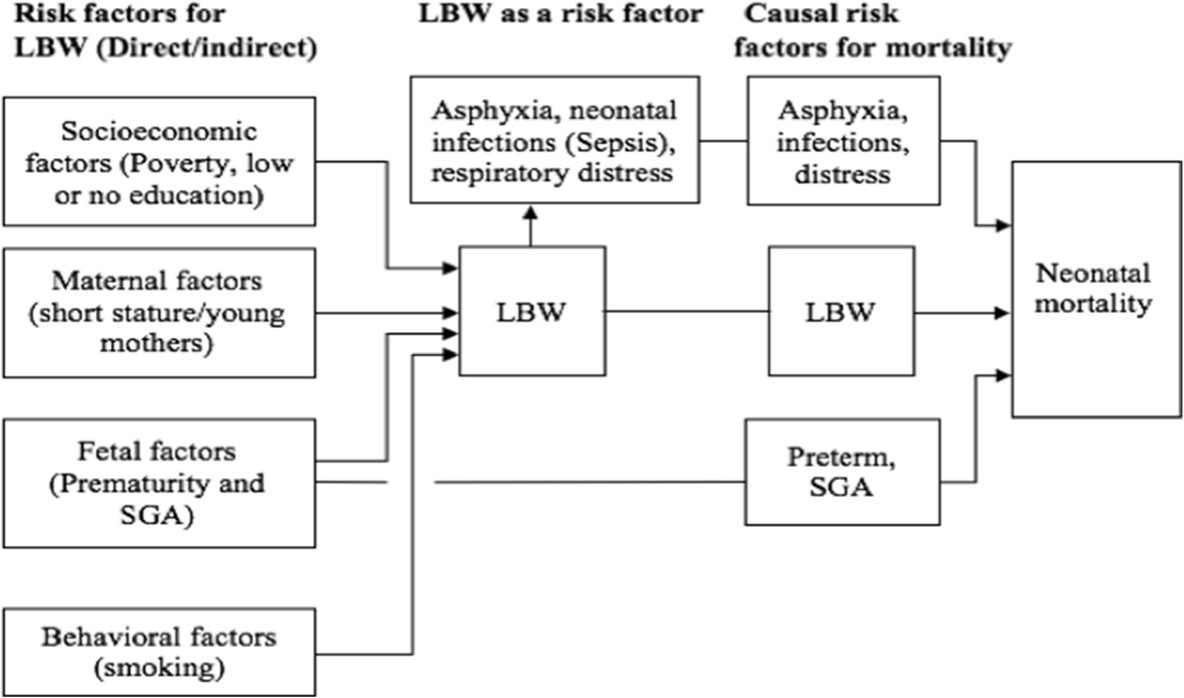
Conceptual visualization of potential risk factors leading to LBW and neonatal mortality. LBW – Low birthweight, SGA – Small for gestation age

In this study, independent variables that are known to be direct and indirect risk factors for neonatal mortality and LBW such as ‘young’ maternal age (7) and poor nutrition (resulting from poverty and low or no education (9) were investigated. Wealth status was determined as a composite cumulative living standard measured in terms of household asset inventory. These were investigated in the univariate analysis to determine their distribution and possible associations with birthweight and neonatal survival categories. Smoking was not examined due to lack of data. Figure 1 below shows a conceptual visualization of LBW as an overriding cause of the majority of neonatal deaths.

Below (Table 1) is a summary of outcome and predictor variables and the covariates that influence the occurrence of low birthweight and the survival of neonates.

**Table 1:** Summary of variables

Variables	Categories	Descriptions
Outcome variable		
Neonatal mortality	Yes (Dead)	Died within age ≤ 1 month
	No (Alive)	Alive at age ≥ 1 month
Predictor variable		
Low birthweight	Yes	< 2500 g
	No	≥ 2500 g ≤ 4000 g
Maternal and socio-economic variables		
Maternal age	< 20 years	
	20–34 years	
	35–49 years	
Wealth status	Poor	
	Middle/rich	
Maternal education	No education	No formal education
	Primary	< 9 years of education
	Secondary/higher	≥9 years of education
Parity	Primiparous	First ever birth
	Para 2–3	2–3 children
	Para 4+	4 or more children
Marital status	Single	Never married, widowed, separated/divorce at delivery time, not living with the spouse
	Married	Married or cohabiting
Place of residence	Rural	
	Urban	
Cesarean birth	No	
	Yes	
Check-up for pregnancy complications	No	
	Yes	

### Data analysis

We used analytical software IBM SPSS version 24 and MS excel for analyses. Pearson’s chi square test of independence and association was used to examine the distribution of variables according to birthweight and neonatal mortality for each survey. Survival plots of the birthweight categories were generated using Kaplan-Meier’s estimator. Binomial logistic regression analysis was used to determine the odds ratios for the association between LBW and neonatal mortality after adjusting for socio-demographic and maternal factors, cesarean births and check-ups for pregnancy complications. The analysis was conducted at 5% significant level. In order to improve the validity of the results, the national representativeness of the data and to adjust for non-response, the complexity of DHS sampling design was taken into account, and data sampling weights were applied to datasets for the years 2000−2001, 2006, and 2011. However, the 1995 dataset was not subjected to weighting due to the need to maintain the statistical power of the data for that year, the implication of which is a very minimal difference. A total of 5973 last-born live births with birthweights were included in the analyses.

### Estimation of LBW-attributable mortality risk fraction among LBW neonates and in the population

The LBW-attributable neonatal mortality risk fraction (AF) and population-attributable mortality risk fraction (PAF) were computed as proportion of prevalent deaths that could be avoided if LBW was prevented or the death of LBW babies was eliminated. These were calculated manually using eqs. (1) and (2) below.

1 $$ \mathrm{AF}=\left(\frac{OR-1}{OR}\right)\ast 100, $$

The population attributable mortality risk fraction PAF, expressed as a percentage (%) was computed using the eq. (2).

2 $$ \mathrm{PAF}={{\mathrm{P}}_{\mathrm{e}}}^{\ast}\mathrm{AF}={\mathrm{P}}_{\mathrm{e}}\ast \left(\frac{OR-1}{OR}\right)\ast 100, $$

OR is the odds ratio generated from binary logistic regression analysis and *Pe* is the proportion of deaths that have the exposure.

## Results

Table 2 shows birthweight and maternal and socio-demographic characteristics of last-born live births by neonatal survival status in Uganda. Overall, the average proportion of neonatal deaths among LBW babies between 1995 and 2011 was about 3.5% while the average proportion of neonatal deaths among normal weight babies (≥2500 g ≤ 4000 g) during the same period was less than 1 %. Cesarean birth was associated with neonatal mortality only in the year 2000−2001 (*p* <  0.05).

**Table 2:** Distribution of birthweight, maternal and sociodemographic characteristics by neonatal survival status in Uganda, 1995–2011

Variables	1995			2000–2001			2006			2011		
	Survival, *N* = 1160			Survival, *N* = 1100			Survival, *N* = 1514			Survival, *N* = (2199)		
	Died n (%)	Lived n (%)	*P* value	Died n (%)	Lived n (%)	*P* value	Died n (%)	Lived n (%)	*P* value	Died n (%)	Lived n (%)	*P* value
Birthweight												
< 2500 g	4 (3.3)	118 (96.7)	< 0.01	5 (4.6)	104 (95.4)	< 0.01	5 (2.8)	175 (97.2)	< 0.05	7 (2.9)	234 (97.1)	< 0.05
≥ 2500 g	6 (0.6)	1032 (99.4)		10 (1.0)	981 (99.0)		11 (0.8)	1323 (99.2)		22 (1.1)	1936 (98.9)	
Maternal age												
< 20	1 (0.6)	155 (99.4)	> 0.05	1 (0.9)	111 (99.1)	> 0.05	2 (1.4)	138 (98.6)	> 0.05	2 (1.3)	154 (98.7)	> 0.05
20–34	6 (0.7)	855 (99.3)		12 (1.4)	825 (98.6)		11 (1.0)	1105 (99.0)		15 (1.0)	1496 (99.0)	
35–49	3 (2.1)	140 (97.9)		3 (2.0)	148 (98.0)		2 (0.8)	254 (99.2)		7 (1.6)	427 (98.4)	
Wealth index	*n* = 392^b^			*n* = 424^b^								
Poor	1 (0.7)	137 (99.3)	> 0.05	1 (0.5)	187 (99.5)	> 0.05	4 (0.9)	442 (99.1)	> 0.05	7 (1.1)	652 (98.9)	> 0.05
Middle / Rich	4 (1.6)	250 (98.4)		3 (1.3)	233 (98.7)		11 (1.0)	1056 (99.0)		17 (1.1)	1426 (98.9)	
Maternal education												
No education	2 (1.5)	132 (98.5)	> 0.05	2 (1.6)	124 (98.4)	> 0.05	3 (1.6)	179 (98.4)	> 0.05	2 (1.2)	171 (98.8)	> 0.05
Primary	6 (0.9)	653 (99.1)		8 (1.6)	605 (98.4)		7 (0.8)	857 (99.2)		12 (1.0)	1149 (99.0)	
Secondary higher	2 (0.5)	365 (99.5)		5 (1.4)	356 (98.6)		5 (1.1)	462 (98.9)		11 (1.4)	757 (98.6)	
Parity												
Primiparous	3 (1.0)	296 (99.0)	> 0.05	4 (1.4)	278 (98.6)	> 0.05	6 (1.7)	356 (98.3)	< 0.05	3 (0.7)	424 (99.3)	> 0.05
Para 2–3	3 (0.6)	532 (99.4)		5 (1.0)	483 (99.0)		7 (1.1)	622 (98.9)		11 (1.2)	945 (98.8)	
Para 4+	4 (1.2)	322 (98.8)		6 (1.8)	323 (98.2)		2 (0.4)	520 (99.6)		10 (1.4)	709 (98.6)	
Marital status												
Single	1 (0.5)	199 (99.5)	> 0.05	2 (1.0)	198 (99.0)	> 0.05	2 (0.7)	277 (99.3)	> 0.05	3 (0.8)	354 (99.2)	> 0.05
Married	9 (0.9)	951 (99.1)		14 (1.6)	887 (98.4)		13 (1.1)	1221 (98.9)		22 (1.3)	1722 (98.7)	
Residence												
Rural	5 (1.0)	517 (99.0)	> 0.05	11 (1.5)	737 (98.5)	> 0.05	10 (0.9)	1051 (99.1)	> 0.05	17 (1.1)	1493 (98.9)	> 0.05
Urban	5 (0.8)	633 (99.2)		4 (1.1)	348 (98.9)		5 (1.1)	447 (98.9)		7 (1.2)	584 (98.8)	
Delivery mode												
Cesarean	1 (1.4)	71 (98.6)	> 0.05	4 (4.4)	87 (95.6)	< 0.05	1(0.8)	122 (98.2)	> 0.05	5(2.1)	230 (97.9)	> 0.05
Normal	9 (0.8)	1079 (99.2)		12 (1.2)	995 (98.8)		14 (1.0)	1372 (99.0)		24 (1.2)	1940 (98.8)	
Check-up^a^												
No	No data			11 (1.5)	742 (98.5)	> 0.05	6 (0.7)	866 (99.3)	> 0.05	13 (1.5)	843 (98.5)	> 0.05
Yes				4(1.2)	332 (98.2)		9 (1.4)	613 (98.6)		14 (1.1)	1261 (98.9)	

*P* values were generated from Chi square analysis*.* Statistical significance (*p* < 0.05, two-sided)

^a^complications

^b^The separate totals(n) for wealth index in 1995 and 2000 shows a deviation from the total (N) due to missing data

Table 3 shows the distribution of the study variables by birthweight. Statistical significantly higher proportions (*p* <  0.05) of mothers with no formal education had LBW babies in almost all the years except 2011. Similarly, maternal age < 20 years of age was associated with having higher proportions of LBW babies as shown in the 1995 and 2006 findings (*p* < 0.01).

**Table 3:** Univariate analysis of maternal and sociodemographic characteristics of neonates by birthweight in Uganda, 1995–2011

Variables	1995, *N* = 1160			2000–2001, *N* = 1100			2006, *N* = 1514			2011, *N* = 2199		
	LBW (%)	NBW (%)	*P* value	LBW	NBW	*P* value	LBW	NBW	*P* value	LBW	NBW	*P* value
Maternal age												
< 20	26(16.7)	130(83.3)	< 0.01	15(13.4)	97(86.6)	> 0.05	27(19.1)	114(80.9)	< 0.01	20(12.7)	137(87.3)	> 0.05
20–34	81(9.4)	780(90.6)		77(9.2)	761(90.8)		112(10.0)	1004(90.0)		174(11.5)	1337(88.5)	
35–49	15(10.5)	128(89.5)		17(11.2)	135(88.8)		41(16.0)	216(84.0)		39(9.0)	395(91.0)	
Wealth	*n* = 392			*n* = 424^a^								
Poor	15(10.9)	123(89.1)	> 0.05	19(10.1)	169(89.9)	> 0.05	61(13.7)	385(86.3)	> 0.05	72(10.9)	587(89.1)	> 0.05
Middle/rich	26(10.2)	228(89.8)		25(10.6)	211(89.4)		118(11.1)	949(88.9)		161(11.2)	1282(88.8)	
Education level												
No education	24(17.9)	110(82.1)	< 0.01	21(16.7)	105(83.3)	< 0.01	29(15.9)	153(84.1)	< 0.05	27(15.6)	146(84.4)	> 0.05
Primary	67(10.2)	592(89.8)		60(9.8)	555(90.2)		101(11.7)	763(88.3)		121(10.4)	1040(89.6)	
Secondary	31(8.4)	336(91.6)		28(7.8)	332(92.2)		49(10.5)	418(89.5)		85(11.1)	684(88.9)	
Parity												
Primiparous	45(15.1)	254(84.9)	< 0.01	27(9.6)	255(90.4)	> 0.05	50(13.8)	312(86.2)	> 0.05	58(13.6)	368(86.4)	> 0.05
Para 2–3	48(9.0)	487(91.0)		51(10.5)	437(89.5)		69(11.0)	560(89.0)		98(10.3)	858(89.7)	
Para 4+	29(8.9)	297(91.1)		31(9.4)	300(90.6)		60(11.5)	462(88.5)		77(10.7)	643(89.3)	
Place of residence												
Rural	67(12.8)	455(87.2)	< 0.05	76(10.1)	674(89.9)	> 0.05	134(12.6)	928(87.4)	> 0.05	167(11.1)	1343(88.9)	> 0.05
Urban	55(8.6)	583(91.4)		33(9.4)	319(90.6)		46(10.2)	406(89.8)		66(11.1)	526 (88.9)	
Marital status												
Single	25(12.5)	175(87.5)	> 0.05	29(14.5)	171(85.5)	< 0.05	39(13.9)	241(86.1)	> 0.05	36(10.1)	321(89.9)	> 0.05
Married	97(10.1)	863(89.9)		80(8.9)	821(91.1)		141(11.4)	1093(88.6)		197(11.3)	1547(88.7)	
Cesarean												
Yes	4(5.6)	68(94.4)	> 0.05	11(11.8)	82(88.2)	> 0.05	24(19.7)	98(80.3)	< 0.01	29(12.3)	206(87.7)	> 0.05
No	118(10.8)	970(89.2)		99(9.8)	909(9.2)		154(11.1)	1232(88.9)		212(10.8)	1752(89.2)	
Check-up												
No	No data			72(9.5)	683(90.5)	> 0.05	105(12.0)	767(88.0)	> 0.05	87(10.2)	769(89.8)	> 0.05
Yes				34(10.1)	302(89.9)		73(11.8)	1315(88.1)		143(11.2)	1132(88.8)	

LBW refers to low birthweight (< 2500 g), NBW refers to normal birthweight (≥2500 g – 4000 g). *P* values were obtained from chi square test

^a^The separate totals (n) for wealth index in 1995 and 2000 shows a deviation from the total (N) due to missing data

In all surveys, LBW was significantly associated with neonatal mortality as shown in Table 4 below. The adjusted odds ratio (AOR) for the years in question were as follows: in 1995, 6.2 (95% CI (2.3 − 17.0), in 2000−2001, 5.3 (95% CI 1.7 − 16.1), in 2006, 4.3 (1.3 − 14.2), and in 2011, 3.8 (95% CI 1.3 − 11.2). The 1995 and 2000–2001 data were not adjusted for wealth status due to large amounts of missing data. Birth complications were also not adjusted for in 1995 due to absence of data.

**Table 4:** Logistic regression analysis showing association between low birthweight and neonatal mortality in Uganda, 1995 − 2011

	Adjusted odds ratios (95% confidence interval)			
Variable	1995	2000−2001	2006	2011
	*N* = 1160	*N* = 1100	*N* = 1519	*N* = 2223
Birthweight				
Low birthweight	6.2 (2.3 − 17.0)^b^	5.3 (1.7 − 16.1)^b^	4.3 (1.3 **−** 14.2)^a^	3.8 (1.3 **−** 11.2)^a^
Normal birthweight	1.0	1.0	1.0	1.0

LBW refers to low birthweight < 2500 g, NBW refers to normal birthweight (≥2500 g – 4000 g)

^a^Adjusted for all socio-demographic, maternal, pregnancy and birth related factors in Table 1

^b^ Adjusted for all socio-demographic (except wealth status), maternal, pregnancy and birth related factors in the study (Table 1). Complications were not adjusted for in 1995

**Fig. 2:**
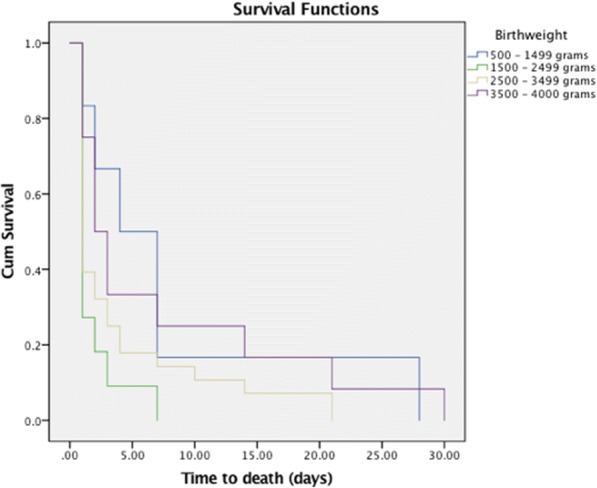
Kaplan-Meier survival curves by birthweight for neonates in Uganda between 1995 and 2011. Cum - cumulative

Figure 2 below shows the relationship between birthweight and time-to-death among neonatal mortality cases, combining all the study years. In conjunction with the survival table (not included in the paper), we observed that over 85% of all neonatal deaths in our study sample occurred in the first week of life. About 95% of all the LBW (< 2500 g) neonatal deaths occurred within the first week of life. In comparison, about 82% of deaths among neonates with NBW (2500 g ≤ 4000 g) took place within in the first weeks. The rest died later, in the second, third, and fourth weeks. The figure also shows an inverse proportionality relationship between weight and survival. With the exception of an outlier, the neonates with higher birthweights tended to survive longer, i.e. beyond the first week.

The LBW-attributable neonatal mortality in Uganda declined by more than half, from 33.6% (%) in 1995 to 15.3% in 2011 as shown in Table 5 below. Similarly, LBW-attributable neonatal mortality among LBW babies also declined by 10.2% from 83.9% to 73.7% in the same period.

**Table 5:** Low birthweight-attributable neonatal mortality risk proportions in Uganda between 1995 and 2011

	Year of survey	Attributable risk fraction (%)
Among LBW neonates (AF)	1995	83.9
	2000*–*2001	81.1
	2006	76.7
	2011	73.7
In the entire population (PAF)	1995	33.6
	2000*–*2001	27.0
	2006	24.0
	2011	15.3

*LBW* low birthweight, *AF* Attributable Fraction, *PAF* Population Attributable Fraction

**Fig. 3:**
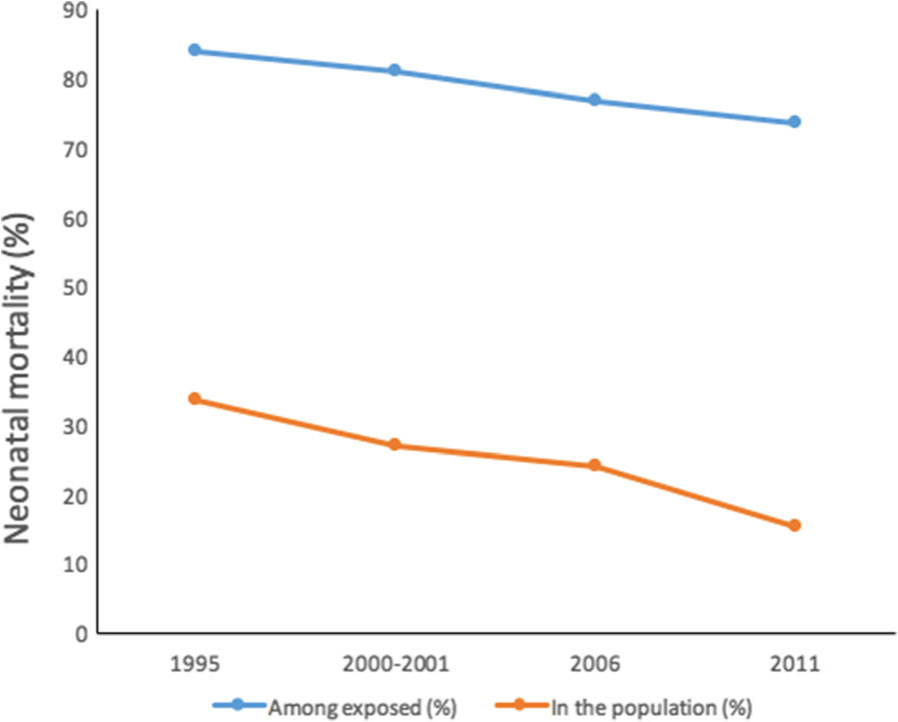
Graphical representation of low birthweight-attributable neonatal mortality trends in Uganda between 1995 and 2011

Figure 3 shows a non-uniform but continuous decline of LBW-attributable neonatal mortality in Uganda between 1995 and 2011.

## Discussion

Overall, the odds of neonatal mortality among LBW babies as compared to normal birthweight were reduced by a third, from about 6 times higher in 1995 to 3.8 times higher in 2011. The LBW-attributable neonatal mortality in the population declined by more than half, from 33.6% in 1995 to 15% in 2011. This present study is the first of its kind in Uganda and perhaps the whole of east Africa that examines the trends of LBW-attributable mortality over the years. The study reinforces the very few LBW-related studies in Uganda and east Africa by providing new peer-reviewed findings on the contribution of LBW towards neonatal mortality countrywide over a period of over 15 years. The study findings might be useful for auditing the causes of neonatal deaths, and for evaluation, future health planning and policy making aimed at improving neonatal survival. The WHO emphasizes that auditing the causes of neonatal deaths is paramount for effective monitoring and improving mother and child health care [37].

The 3.8 times higher odds of deaths among LBW neonates in 2011 in the present study is consistent with the findings of a related study conducted by Kananura et al. in eastern Uganda in 2012–2013 that indicated a 3.51 mortality odds ratio [36]. Comparable findings were also obtained in a follow-up study in western Uganda, completed in 2006 but analyzed by Marchant et al. in 2012 [15]. This study estimated the odds of neonatal mortality among LBW newborns relative to NBW newborns at 3.45 [15]. Our findings of 15.3% LBW-attributable neonatal mortality in 2011 in the population are comparable to the findings of a situation analysis study conducted by the Ministry of Health (MoH) in Uganda in 2008 [38]. The MoH study combined both quantitative and qualitative methods and collected data from 10 districts covering the four conventional regions (Central, Eastern, Western and Northern) in Uganda. In this MoH study, the health personnel interviewed about perinatal outcomes in the health units indicated that LBW contributed to 16% of the total newborn deaths [38]. However, the study also acknowledged the underreporting of LBW as a cause of death due to overlaps with infections and breathing difficulties [38].

The results indicated a significantly higher proportion of deaths among LBW babies and this corroborates with findings of other studies [2, 3] that show higher mortalities among LBW newborns relative to their NBW counterparts. Although cesarean births have been associated with mortality as also shown by the findings (*p* < 0.05) for the year 2000–2001 in Table 2, in 2006 and 2011 however, the findings (*p* >  0.05) indicated improvements in obstetric services that has enabled the survival of many cesarean birth babies.

Figure 2 showed that about 85% of neonatal deaths occurred in the first week after birth. This is close to the estimate of a recent MoH report on maternal, perinatal and child death review that indicated about 75% neonatal deaths in the first week [39]. The inverse proportional relationship indicated by the trends of birthweight versus time-to-death among neonatal deaths in Fig. 2 concurs with findings from a hospital-based study in Dhaka, Bangladesh [40]. The findings in Fig. 2 also implied that the risk of neonatal death is inversely proportional to birthweight and are in agreement with several other studies [40–43]. However, our data on age at death (days) appeared to have been aggregated in terms of 7 days (weekly) and not the actual mortality days. This slightly compromised the accuracy of the Kaplan Meier’s survival curve in our study in terms of days of survival.

According to a facility-based study by Hedstrom et al. in central Uganda that admitted neonates born between December 2005 and September 2008, 89% of neonatal deaths among LBW neonates weighing under 1000 g could be attributable to LBW [43]. Another study by Marchant et al. [15] that utilized data collected in 2006 in western Uganda also estimated a 71% LBW-attributable neonatal mortality among LBW neonates. Both of these findings are comparable with the LBW-attributable mortality estimates among LBW babies in the whole country in this present study.

Neonatal mortality accounts for about 40% of global under-five mortality [44]. In Uganda, in recent years, it was estimated that about 45,000 neonates die every year [20]. By extension of our findings, this corresponds to approximately 7000 (15.3%) neonatal deaths attributable to LBW in 2011. Although our findings could be a slight underestimation given the many unrecorded births (about 45% in 2011) [43] and unregistered neonatal deaths, they provide comparable national estimates that can be used for advocacy and countrywide public health planning to reduce LBW-attributable neonatal deaths. For instance, the successful Kangaroo Mother Care project for premature and LBW newborns initiated by Uganda Newborn Study project (UNEST) in 2007–2011 in Iganga and Mayuge district [45] could be implemented countrywide.

The greatest national decline of LBW-attributable mortality estimated in 2011 in our study is a notable finding that could be attributed to the efforts of the inter-agency national Newborn Steering Committee (NSC) [46]. The NSC, which was initiated in 2006, ensured rapid policy adaptation and implementations both at the health facility and community levels in the few years to 2011 [46]. It was mandated by the MoH to spearhead comprehensive service delivery and community-and health facility-based training [46, 47]. Our findings thus reveal that the policy changes and its implementation may have had a profound positive impact on the survival of LBW newborns during this period. The findings indicate that it is possible to eliminate unnecessary neonatal deaths due to LBW and make significant contributions towards achieving the SDG 3.2 target that aims to lower neonatal death rate to 12 per 1000 live births by 2030 [23]. Further, both the present study findings and the NSC initiative could be of keen interest to similar countries (with high neonatal mortalities) for policy making and study replications with the aim of improving LBW neonatal survival, for instance, in the Philippines, where the decline of neonatal deaths has stagnated [48].

Also, the Uganda Newborn Study (UNEST) Project partly contributed to the decline in mortality of LBW and preterm newborns in parts of eastern Uganda and consequently contributed to the overall national decline during this period [45].

The survival analysis indicated that the rate of decline in LBW-attributable mortality in the 5-year periods increased from 6.6% between 1995 and 2000–2001 to 8.7% between 2006 and 2011 in the population (Table 5). However, between the two periods, there was a significant deceleration in the decline to 3.0% between 2000 and 2001 and 2006 (Fig. 3 and Table 5). This could potentially be due to the 20% decline in the use of family planning methods among < 20 years old sexually active girls during this period as noted by the analytical overview of the Ugandan child report [49]. This could have led to increased teenage pregnancies. LBW are common among teenage mothers (< 20 years) [7] and the mortality among babies born to younger mothers in Uganda was also notably high between 1995 and 2005 [22]. Nevertheless, our findings in Table 2 did not show any significant higher mortality numbers among the < 20 years old mothers, perhaps because of the few number of births in this age-group in our sample selection. However, statistically reasonable numbers in 2006 showed a significant association between primipara mothers (most of whom were younger mothers (Table 3)) and neonatal mortality. A study conducted by Andualem et al. in western Uganda between 2005 and 2008 revealed that over 82% of female students had unmet sexual/reproductive health counseling needs [50]. Lack of knowledge about the signs of pregnancy complications has been linked to birth unpreparedness in Uganda [51], a consequent risk factor for neonatal deaths, including LBW deaths. A comparative development study by Kevin Croke [52] also highlighted the decline in the health system gains in Uganda between 2001 and 2006 due to political shocks related to removal of presidential term limits. Financing of the health care system was negatively affected. This could partly account for the rise in LBW-neonatal deaths during this period. The specialized care of LBW babies requires extra financing compared to NBW. The direct impact of the decline in health system gains on survival of LBW detected by the present study is consistent with WHO/UNICEF observations that survival of LBW neonates, a high-risk infant group, is among the most sensitive indicators to assess the progress of maternal and child health status in a country [2].

There was no statistically significant association between place of residence, maternal education, marital status, wealth status, maternal age, and neonatal mortality, *(P >  0.05)* (Table 2). Although studies vary in their findings concerning the association between these socio-demographic and maternal factors (including parity) and neonatal mortality [53], many study findings have indicated an association between single motherhood [54], teenage maternal age [55–57], lack of education [56], rural residence [57] and neonatal mortality. A systematic review of 17 studies up to the year 2013 in SSA [55] indicated that socio-demographic and maternal risk factors are much more prevalent among teenage mothers as compared to adult mothers [55]. With the decentralized system in Uganda, further analytical research at the districts or regional levels on the effect of socio-demographic factors on birthweight and neonatal deaths would provide more robust findings for monitoring, policy making and interventions. However, at the national level, comprehensive measurement and recording of birthweight need to be made possible, irrespective of whether a child is born at home or at the hospital. As a national policy driven initiative, the provision of weighing scales to health volunteers and midwives at the community level, even on a shared basis based on proximity and locality, is feasible and could be very effective for monitoring neonatal health countrywide. Apart from improving accuracy on birthweight data collection, the availability of weighing scales could also be a profound campaign tool for lowering LBW incidences by highlighting preventive measures. Affordable and easy to maintain mechanical weighing scales have previously been used at the community level in over 400 villages in western Kenya [58]. Although it was on a small scale, the initiative was profoundly successful, as shown by an increase in the birthweight measurements of newborns of about 54%, from 43% to 97% [58]. The current study could thus give the impetus to communities and local organizations to take initiatives and improve the survival of LBW neonates. Further, as LBW is an underlying cause of 60–80% of all neonatal deaths globally (2,3) and about 15% of neonatal deaths in Uganda (present study 2011 findings), continuous data collection on birthweights that supports research, monitoring, and strategic preventive interventions could be a formidable approach to curbing neonatal deaths and overall health systems strengthening both globally and in Uganda.

Although our study largely indicated no significant associations between cesarean birth, pregnancy complications and neonatal mortality for most of the years, a number of studies have found associations between cesarean births [57, 59], pregnancy complications [59] and neonatal deaths. There were inconsistencies in our findings with regard to the significant associations between socio-demographic factors and LBW across all the study years *(p < or > 0.05)* (Table 3). However, there were higher proportions of LBW babies among teenage and uneducated mothers in all the survey years. Teenage pregnancy was associated with LBW only in 1995 and 2006. These findings corroborate study findings elsewhere in rural India [60] and in several SSA countries [7, 61] that strongly indicate that young maternal age is associated with LBW. A study in Brazil, however, found an association between teenage pregnancy and LBW only when marital partners (an economic factor) were lacking [62].

### Methodological considerations

The random sampling of data across the entire country and the standardized nature of data collection method of the DHS strengthen the external validity of our study and enable global comparability among countries. Weighting the data for the years 2000, 2006 and 2011enabled us to adjust for disproportionate sampling and non-response. This improved the national representativeness and validity of the study estimates. The 1995 dataset was not weighted and the results for that year are slightly less representative. However, the results are still valid, due to the fact that there was only a small difference when weighted and unweighted results of all the other years were compared. The national representativeness of the 1995 data was only dependent on the random sampling across the entire country and the standardized nature of DHS data collection for its reliability.

The repeated findings of significant associations between LBW and neonatal mortality across all surveys confirm the existing evidence of association and the internal validity of this present study. Nonetheless, our study could not confirm the causal association because the exact causes of newborn deaths were not ascertained medically. The in-depth use of the nationally representative DHS datasets in this study has revealed the need to improve data collection techniques and to include other similarly important variables such as diagnostic causes of death among individual children, for example, birth asphyxia.

Another limitation of our study was that although hospital births recorded and/or communicated birthweights, over 65% were from mothers` recall and the rest from the health card, and we cannot therefore completely dismiss the possibility of recall bias. This also applies to the 1995 data that included both hospital and home births. Nevertheless, child birth is a significant event in a mother’s life and with our study selection of the most recent birth experience, there is a very high possibility that the mothers recalled correct birthweights. Moreover, for the years 2000 to 2011, birthweight data concerned solely information regarding hospital born babies because these were measured birthweights and not estimated weights as in-home births, where birthweights are mainly estimated based on the physical size of the body parts such as foot length, chest or head [63]. A study in Uganda compared the accuracy of a proxy measure of LBW by midwives in a hospital-based setting showed an accuracy of over 80%. However, the study also noted the limitation that the findings may not reflect the actual situation in the communities where less skilled community volunteers assist in most births, and their estimates of cut-offs are prone to bias [63]. Elimination of macrosomic newborns improved the validity of our findings.

Although the 1995 data included both home and hospital births, which undermined the consistency of the study methodology across years, preliminary analysis indicated that among the selected sample of newborns with birthweight measures in 1995, only 3.5% of the births were home births (or perhaps on the way to the hospital). The 1995 data thus has a reasonable degree of consistency with other survey years. However, the selection of only hospital births in other survey years improved the quality and validity of the findings for those years.

The recording of neonatal survival data from day 0 to 30 by the DHS allowed us to clearly categorize our outcome variable and investigate risk factors across all the survey years with consistency. Given the large number of home births (about 50%) in all the surveys, both the LBW and neonatal deaths were likely underreported.

The birthweight data are prone to rounding-off or aggregation into 500 g-weight intervals which could have slightly compromised the accuracy of Kaplan-Meier’s survival analysis in this study. This aggregation of data was observed in a study by Channon et al. [64]. However, the fact that over 90% of LBW neonatal deaths in our study occurred in the first week is quite consistent with global WHO findings that 75% of neonatal deaths occur in the first week [65], given the high-risk group of LBW in a low-income country.

## Conclusion

Low birthweight is associated with neonatal mortality and contributes to a substantial proportion of neonatal deaths in Uganda. Although significant progress has been made to reduce newborn deaths attributed to LBW, by 2011, about 74% of all LBW neonates died in the neonatal period. This implies that the health system in place has been inadequate to meet the challenge of ensuring LBW survival. There is also profound need to strengthen both birth and neonatal death registration irrespective of whether the infants are born at home or at the health centers. The decentralized health system in Uganda can enable community health workers (CHW) and the village health teams (VHT) in liaison with the sub-counties and the districts to close the existing gaps concerning neonatal birth and death audits. This will enable robust and continuous research and monitoring of the progress of LBW neonatal survival. Our study presents national estimates of risks and mortality trends that provide national basis for continual evaluation and policy recommendations to prevent LBW and minimize risks of neonatal deaths. A holistic approach to reduce the incidence of preventable LBW babies could be fostered to reduce these mortality rates. Viable fronts that could be strengthened include sexual education in schools to prevent teenage pregnancies, complementing nutritional diet of pregnant mothers, HIV testing, ensuring that all pregnant mothers use mosquito nets, training of health workers, and promoting antenatal care visits and hospital births. Enhancing the quality of postnatal care could also reduce mortality incidence of LBW newborns.

## Abbreviations

AF: Attributable fraction
AOR: Adjusted odds ratio
CHW: Community health workers
DHS: Demographic and health survey
EA: East Africa
LBW: Low birthweight
MDG: Millennium development goals
MoH: Ministry of Health
NBW: Normal birthweight
NSC: Newborn Steering Committee
PAF: Population attributable fraction
SDG: Sustainable development goal
SGA: Small for gestational age
SSA: Sub-Saharan Africa
UNAP: Uganda nutrition action plan
UNEST: Uganda newborn study
VHT: Village health teams
